# Effects of estradiol and medroxyprogesterone acetate on morphology, proliferation and apoptosis of human breast tissue in organ cultures

**DOI:** 10.1186/1471-2407-6-246

**Published:** 2006-10-18

**Authors:** Natalija Eigėlienė, Pirkko Härkönen, Risto Erkkola

**Affiliations:** 1 Department of Obstetrics and Gynecology, Turku University Central Hospital, 20520 Turku, Finland; 2 Department of Anatomy, Institute of Biomedicine, University of Turku, 20520 Turku, Finland; 3 Kaunas University of Medicine, 44307 Kaunas, Lithuania; 4 Department of Laboratory Medicine, Lund University, MASUniversity Hospital, CRS, 20502Malmö, Sweden

## Abstract

**Background:**

Human breast tissue undergoes phases of proliferation, differentiation and regression regulated by changes of the levels of circulating sex hormones during the menstrual cycle or aging. Ovarian hormones also likely play a key role in the etiology and biology of breast cancer. Reports concerning the proliferative effects of steroid hormones on the normal epithelium of human breast have been conflicting. Some studies have shown that steroid hormones may predispose breast epithelial cells to malignant changes by stimulating their proliferation, which is known to be regulated tightly by stromal cells.

The aim of this study was to investigate the effects of 17β-estradiol and medroxyprogesterone acetate on proliferation, apoptosis, expression of differentiation markers and steroid hormone receptors in breast epithelium using an *in vitro *model of freshly isolated human breast tissue, in which a proper interaction of breast epithelium and stroma has been maintained.

**Methods:**

Human breast tissues were obtained from women undergoing surgery for breast tumours. Peritumoral tissues were excised and explants were cultured for 3 weeks in medium supplemented with E_2 _or MPA or with E_2_+MPA. Endpoints included histopathological, histomorphometric and immunohistochemical assessment of the breast explants.

**Results:**

Culture of breast explants for 14 or 21 days with steroid hormones increased proliferative activity and the thickness of acinar and ductal epithelium. E_2_-treatment led to hyperplastic epithelial morphology, MPA to hypersecretory single-layered epithelium and E_2_+MPA to multilayered but organised epithelium.

The proliferative response to E_2 _in comparison to control *(p < 0.001) *was more pronounced than to MPA *(p < 0.05) *or E_2_+MPA *(p < 0.05) *at 7 and 14 days for Ki-67 and PCNA. E_2 _treatment also decreased the proportion of apoptotic cells after 7 *(p < 0.01) *and 14 *(p < 0.01) *days. In addition, the relative number of ERα, ERβ and PR positive epithelial cells was decreased by all hormonal treatments.

**Conclusion:**

Organ culture system provides a model for studying the direct effects of steroid hormones and their analogues on postmenopausal human breast tissue.

Addition of E_2 _or MPA or E_2_+MPA to breast explants caused characteristic changes in morphology, stimulated epithelial proliferation, lowered apoptosis ratio and decreased the relative number of epithelial cells expressing ERα, ERβ and PR.

## Background

Breast cancer is the most common malignancy among women especially in developed countries [1]. Its incidence strongly correlates with age and rises steadily to reach a peak in the postmenopausal age. Simultaneously, post menopause is associated with low levels of ovarian steroids leading to unwanted climacteric symptoms and illnesses, such as vasomotor symptoms, mood and sexual dysfunction, osteoporosis and bone fractures.

Estrogen-alone or in combination with progestins has been widely used for hormone replacement therapy (HT) for prevention and treatment of these conditions [2]. Despite the benefits of HT, some studies have shown that HT may increase the risk of breast cancer. One of the mechanisms suggested for carcinogenesis is that endogenous estrogens and progesterone, as well as their synthetic and/or exogenously administered derivates influence cell proliferation in breast glandular tissue [3,4]. Stimulation of proliferation predisposes epithelial cells to occurrence of somatic mutations and eventually to malignant changes [5], especially during long-term HT [6,7]. However, recent results from WHI suggested that treatment with estrogen-alone does not increase breast cancer incidence in postmenopausal women with prior hysterectomy [8].

Conflicting results have been obtained from *in vitro *and *in vivo *studies concerning the role of estrogen and especially of progestins in breast epithelial cell proliferation and in breast carcinogenesis [9].

*In vivo *the proliferative activity of breast epithelial cells is highest during luteal phase of menstrual cycle coinciding with high serum estrogen and progesterone levels [10-14]. On the other hand, *in vitro *studies on breast cell lines have shown that estrogen increases cell proliferation, but addition of progesterone has an antiproliferative effect [15]. Thus, further clarification of the effects of HT on normal human mammary gland is required [16].

The optimal method for estimation on the effects of HT on normal breast epithelium is a large-scale clinical study in a normal human population. However, these studies take years and need great numbers of healthy volunteers. On the other hand, studies using cell lines and experimental animals (*e.g*. rodents), although helpful, have their limitations [17] due to lack of epithelial-stromal interaction in cell lines or the biological differences between human and rodent mammary glands [16]. However, there are also some studies on macaque's mammary glands, which have well-documented similarities to the anatomy of human mammary gland, reproductive physiology and peripheral steroid hormone metabolism [18-20]. These studies are of great value, but they are expensive, taking months or years and need animal sacrifice.

Experimental models have shown the importance of epithelial-stromal interaction for proliferation and differentiation of epithelial cells both *in vivo *and *in vitro *[21-25]. The advantage of tissue culture over cell lines lies in the maintenance of the three-dimensional structure of the tissues and in the maintenance of epithelial-stromal interactions. There is evidence for tissue-specific hormonal effects to be maintained more properly in tissue culture when compared to primary culture models [21-25].

The human breast culture provides a useful *in vitro *model to monitor the response of the breast tissue to various HTs. There have been reports of human breast organ cultures *in vitro *[26-29], but they have been mainly morphological studies and only a few of them have included data on regulation and expression of steroid hormone receptors [16]. Normal glandular tissue can be obtained from three different types of procedure: reductive breast surgery, surgery performed for benign tumours and normal epithelium surrounding the tumour (peritumoral tissue or adjacent to tumour tissue). The main source of normal breast tissue for culturing *in vitro *is reductive mammoplasties, which are mostly performed on women in reproductive age.

The aim of this study has been to evaluate the changes caused by 17β-estradiol (E_2_) and medroxyprogesterone acetate (MPA) on the postmenopausal human mammary gland *in vitro*. E_2 _and MPA are widely used as HT by pre- and postmenopausal women. Therefore, we used peritumoral tissue (non-tumour areas), which wasobtained from postmenopausal patients in breast tumour operations.

In this work, culture conditions for freshly isolated human breast tissues were established and the *in vitro *effects of E_2 _and MPA were studied on breast histology, differentiation markers, expression of steroid hormone receptors, proliferation and apoptosis ratios in cultured breast explants. These results demonstrate that the morphological integrity of human breast explants can be maintained in organ culture up to 3 weeks.

## Methods

### Ethics

The study protocol was approved by Joint Ethical Committee of Turku University and Turku University Central Hospital. An informed written consent was obtained from all patients before the surgery.

### Tissue samples

Human breast tissues were acquired from nine female patients aged 49–64 years, undergoing breast surgery for breast tumours (n = 9). The normal tissue that we used was taken from the border area between normal tissue and excised tissue. Histologically these tissues pieces contained normal epithelium. Tissue were transported to the laboratory in cold, phenol-red free DMEM/F12 medium (GIBCO, England) supplemented with penicillin (100 IU/ml) and streptomycin (100 μg/ml). The cultures were started within 1–2 h after surgery. The tissues were cut in DMEM/F12 medium. First, breast tissue was carefully dissected under a stereo microscope to exclude as much of adipose tissues as possible saving the collagenous connective tissues, because the ducts and lobules are found mostly in the tan collagenous stroma. The collagenous connective tissues were cut with a fine scissors approximately into 2 × 2 × 2-mm^3 ^pieces.

### Organ culture

**Figure 1:**
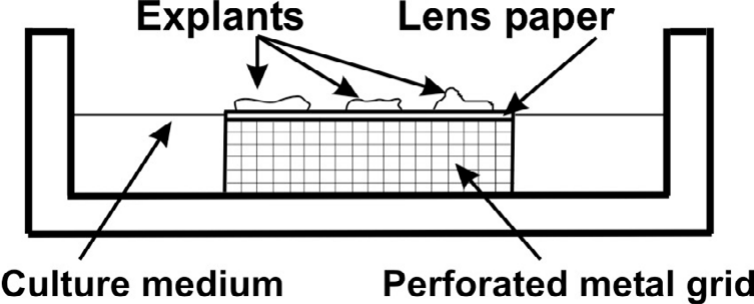
The modified *in vitro *culture method used in the present study.

The culture method of Trowell was used with some modifications [24-26]. Four to seven pieces of breast explants were transferred onto lens papers lying on stainless steel grids in Petri dishes and cultured for different periods (Fig. 1). The explants were kept in a humidified atmosphere with a mixture of 5% CO_2 _and 95% air at 37°C in phenol-red free DMEM/F12 medium (GIBCO, Paisley, UK) supplemented with 10% dextran-charcoal stripped fetal calf serum, penicillin (100 IU/ml), streptomycin (100 μg/ml), 0.5 ml ITS supplement (Sigma, Steinheim, Germany), 100 nM hydrocortisone (Sigma) and 10 ng/ml EGF (Sigma). This medium was referred to as basal medium. 10 nM E_2 _(Sigma), 100 nM MPA (Sigma) or a combination of 10 nM E_2 _and 100 nM MPA was added to the basal medium. These concentrations of steroid hormones were used according to previous study [16]. The solubility of MPA in ethanol was poor, therefore all steroids were dissolved in dimethylsulfoxide (DMSO, Sigma), in which many biologically active compounds have a high solubility. Control tissues were cultured in basal medium, supplemented with solvent (DMSO). The medium was changed every second day and fresh batches of steroids were added. Two to three parallel dishes with samples obtained from each patient were cultured for every treatment group and every time point.

The final concentration of DMSO in the culture medium was 0.03 %. Explants were collected 7, 14 and 21 days after the beginning of the culture.

### Histology

Non-cultured (day 0) and cultured mammary gland tissues were fixed in 4% paraformaldehyde in phosphate-buffer overnight (o/n) at +4°C, then dehydrated, and embedded in paraffin according to routine procedures. Paraffin sections of 5 μm were cut from each piece and stained with hematoxylin and eosin (H&E) for histological examination and from same paraffin blocks, sections were cut for immunohistostainings.

### Immunohistochemistry

For immunohistochemical staining, 5 μm thick paraffin sections were mounted on Super-Frost Plus (Menzel-Gläser, Germany) slides. All primary antibodies used in this study were commercially available mouse primary anti-human antibodies (Table 1). After dewaxing in xylene and rehydrating through graded alcohol series followed by water, endogenous peroxidase in the sections was blocked by immersion for 10 min in a solution of 3% H_2_O_2 _and 100% methanol. The slides were treated for antigen retrieval by boiling in a microwave oven in 0.01 M citrate buffer and 0.5% Tween 20 (pH = 6.0) for 15 min. After washing with PBS (pH 7.4), the sections were incubated with 2% normal horse serum for 30 min at RT to block non-specific binding. Then sections were incubated with primary antibodies at different dilutions o/n at +4°C. PBS was used for all subsequent washes and for dilution of the antibodies. The slide detected positive for antigen was used as positive control, and the slide incubated without primary antibody was used as negative control for each staining batch. After a further wash step, biotinylated horse anti-mouse secondary antibody (1:200, Vector, USA) was applied for 2 hours at RT. Visualization was carried out by means of the avidin-biotin peroxidase complex (ABC, Vector, USA) for 1 hour, followed by diaminobenzine tetrahydrochloride (DAB, Vector, CA) as chromogen in distilled water. Finally, all sections were counterstained by Mayer's hematoxylin for 14 sec., dehydrated and mounted with Mountex (HistoLab, Göteborg, Sweden) mounting medium.

### Image analysis and histomorphometry

The sections were interpreted under light microscopy in high-power fields. An Olympus BX-60 (Olympus Optical Co. GmbH Hamburg, Germany) microscope connected to a computer using analysis Soft Imaging 3.00 (Build 417) System GmbH was used for the image analysis.

Histomorphometric evaluation was performed to estimate the changes of epithelial morphology (acinar and ductal wall thickness) during different hormonal treatments. The same sections assessed for histological examination (H&E-stained slides) were analyzed for histomorphometry. All morphometric measurements were done on H&E-stained slides, using OsteoMeasure™ system (OsteoMetrics, Inc., Atlanta, USA). Morphometric measurements of ducts and acini were performed with the help of digitizing interactive video overlay drawing system run by this morphometry program. The acinar and ductal profiles were measured by outlining their images on the monitor screen with the computer mouse. In each sample, eight microscopic fields (four for ducts and four for acini) were randomly selected and examined at ×200 magnification. The breast structures were quantified by digital tracing, and the wall thickness (μm) of ducts and acini were measured with exclusion of the lumens (Fig. 3A,B).

### Fragment end labelling of DNA (FragEL™)

A DNA fragmentation assay (Klenow FragEL™ DNA Fragmentation Detection Kit, Calbiochem, Germany) was used to detect apoptotic cells in paraffin-embedded tissue sections. All procedures were carried out according to manufacturer's instructions. Briefly, after deparaffination and rehydration of tissue sections in graded alcohol, the sections were subjected to proteinase K treatment for 20 min at RT to enhance the sensitivity of the DNA end labelling. The endogenous peroxidase activity was blocked by incubating the slides in 0.3% hydrogen peroxide in water for 10 min at RT. Biotin-labelled deoxynucleotides were catalytically added to 3'-OH groups at the ends of DNA fragments by Klenow enzyme (DNA polymerase I) during a 90-min incubation at 37°C. Nucleotides incorporated into fragmented DNA were detected after incubation with streptavidin-horseradish peroxidase conjugate (30 min at RT) followed by visualization with 3,3-diaminobenzidine (4 min. at RT) as a chromogen and methyl green as a counterstain. Negative controls were obtained by omitting Klenow enzyme from the reaction buffer. Cells were counted as apoptotic only if they were FragEL-positive (brown) and showed characteristic morphological features typical of apoptosis (cells containing pyknotic nuclei and apoptotic bodies). FragEL-index or apoptosis ratio was defined as the number of FragEL-positive cells (brown) per 1000 epithelial cells counted (brown+green) in sections representing each explant in every culture.

### Quantification of immunohistochemical stainings

All immunostainings were quantified in specific breast structures by counting the proportion of immunostained cells in different compartments (inter- and intralobular ducts or terminal ductules and lobules) of breast tissue. Only normal areas of breast tissues were evaluated. Areas with fibrocystic changes, hyperplasia, microcalcifications, and metaplasia were excluded from the analysis. The expression of all markers was evaluated only in glandular epithelium – luminal epithelial cells (LEC) and myoepithelial cells (ME). The immunostainings were evaluated according staining percentage of the positive cells (Ki67, PCNA, ER, PR) and staining intensity in nucleus (Ki67, PCNA, ER, PR) or cytoplasm (CK8&18, K-14, SMA).

#### The percentage of positive cells

Different cut off percentages of cells with positive immunostaining have been chosen for various markers. For Ki-67 staining, the explants with over 1% of the nuclei being stained were considered positive and for PCNA staining the explants with over 5%. The fraction of immunopositive nuclei relative to all nuclei of cells (staining frequency) determined for 1000 epithelial cell nuclei for each sample to obtain a reliable estimate of the PCNA or Ki-67 proliferation index [30,31].

A specimen was considered positive for ER or PR if staining of ≥10% of nuclei of epithelial cells was seen [32]. For quantification of ER or PR immunostainings an eyepiece grid of 10 × 10 squares at ×200 magnification was used and the positive epithelial cells crossing 100 consecutive squares were counted [33]. Positive cells were counted in extra lobular ducts and terminal-lobular units (TDLU), consisting of acini and small ducts. Numbers of squares, which contained one or more positive cells, were expressed as a percentage of the total number of examined squares.

#### Immunostaining intensity

The mean staining intensity was estimated and expressed as *weak *(yellow or light-brown cytoplasmic or nuclear staining), *moderate *(dark-yellow or medium-brown staining, or a mixture of light and dark staining) or *strong *(dark-brown staining in nucleus or cytoplasm).

### Statistical analysis

Statistical analysis was performed using SAS System software version 9.1.3 (General Mixed Model Analysis of Variance). This analysis estimates the correlation of variables (acinus and duct wall thickness, Ki67, PCNA, FragEL, ERα, ERβ, PR) with time (day 7, 14, 21), treatment groups (control, E_2_, MPA, E_2_+MPA) and interaction between day of culture and treatment. The variables obtained from cultured explants were pooled and used for calculation of the means and standard deviations. The first analysis was done separately for each day (3 different days) with treatment (4 different treatments) as the fixed factor and subjects (individual patients) as random. In the second analysis treatment and day and their interaction were fixed factors and subjects were random. Pairwise comparisons between different treatments were adjusted using the Tukey-Kramer method. Assumptions were checked using analysis of residuals. All the results are expressed as means ± SD. *P*-values less than *0.05 *were considered to be significant.

## Results

### Histological examination of cultured human breast tissue

**Figure 2:**
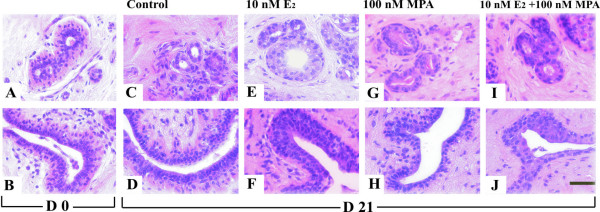
**Histology of human breast before and after organ culture**. The histology analysis of explants revealed differences in the appearance of epithelium before and after organ culture. The acini (**A**) and ducts (**B**) before organ culture (D 0) and after 21 days (D 21) in basal medium (**C-D**); (**E-F**) the epithelium in explants cultured with E_2_; (**G-H**) the epithelium in explants cultured in the presence of MPA; (**I-J**) the epithelium in explants cultured with E_2_+MPA. (H&E staining. Scale bar 100 μm).

Histological examination showed that inter- and intralobular ducts (ducts), terminal ductules and lobules (terminal duct-lobular unit, TDLU) and stroma had retained their morphological structures throughout the culture period of 21 days. Histological analysis of breast tissues revealed differences in the appearance of the epithelium between the different treatment groups (Fig. 2A–J). The lobules (acini) of mammary tissue before culture (D 0) and after 21-days in basal medium (D 21) were small, compact and similar in appearance (Fig. 2A,B). In contrast, in the E_2_, MPA and E_2_+MPA groups, lobules were larger and less compact, the epithelium height was increased. In E_2_-treated explants, the epithelial cells often appeared hyperplastic and in some cases, there was a loss of distinction between duct lining cells and myoepithelial cells. The size of the cells and the number of cell layers was increased. There was also a tendency of epithelium to overgrow and obliterate the basement membrane (Fig. 2E,F). In the explants cultured with MPA the acini were large and the lumina were filled with secretion (Fig. 2G,H). In the explants cultured with E_2_+MPA the number of epithelial cell layers in the ducts was increased (Fig. 2I,J).

### Histomorphometrical analysis of cultured explants

**Figure 3:**
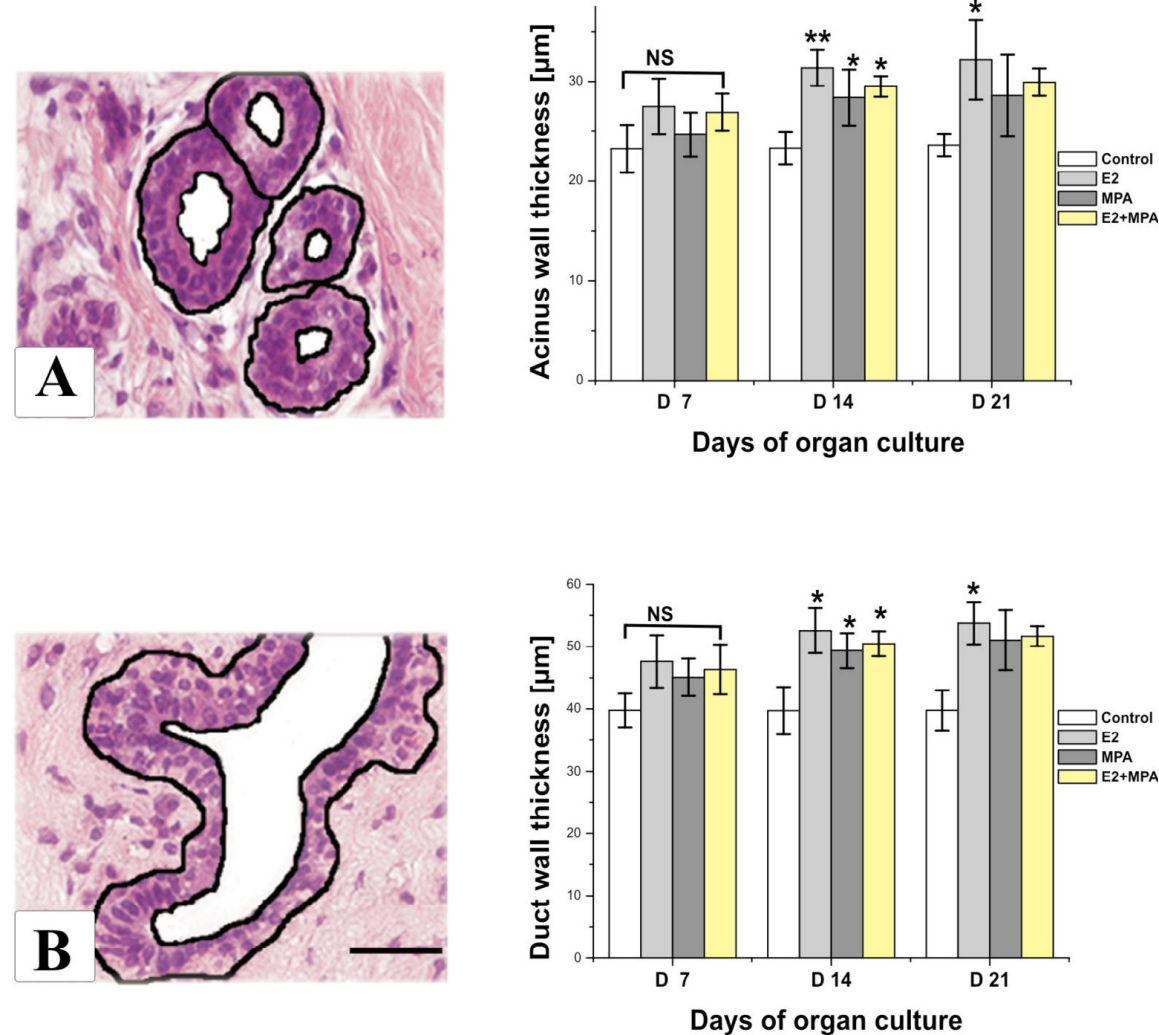
**Histomorphometrical method used to determine acinar and ductal wall thickness in cultured human breast explants**. The areas outlined in black represent the measured sector of acini (**A**) and ducts (**B**). Columns represent the mean counts ± SD. Asterisks indicate the statistical significance of differences between control and treatment groups (*, *p < 0.05*; **, *p < 0.01*) and *NS*, not significant. The differences between treatment groups E_2 _*vs*. E_2_+MPA, E_2 _*vs*. MPA and MPA *vs*. E_2_+MPA did not reach statistical significance at any day of organ culture. (H&E staining. Scale bar 100 μm).

The thickness of acinar and ductal wall was increased by treatment with E_2_, MPA and E_2_+MPA. All measured values (the acinar and ductal wall thickness) were greatest for explants treated with E_2 _for 7, 14 and 21 days, but the differences between the treatment groups (E_2_, MPA and E_2_+MPA) *vs*. control groups at day 7 did not reach statistical significance. After 14 days of culture, there were significant differences in thickness between hormone treatment groups and control, but statistical significance was not observed between hormonal treatment groups – E_2 _*vs*. MPA, E_2 _*vs*. E_2_+MPA or MPA *vs*. E_2_+MPA. After 21 day of organ culture, only the difference between E_2 _and control was statistically significant. The hormone effects on acinar wall thickness were comparable to those on the ductal thickness (Fig. 3).

### Expression of differentiation markers

**Figure 4:**
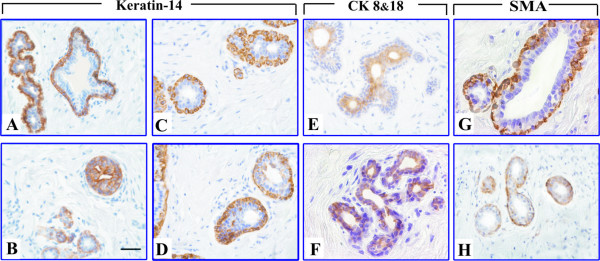
**Immunohistochemistry of differentiation markers in human breast explants**. (**A-B**) Immunohistochemistry of K-14 before organ culture. Note that the myoepithelial cells of the normal tissue have heavy brown cytoplasmic stain (**A**). In tumoral tissue the K-14 positive cells are scattered throughout the acinus (**B**), thus, it does not label anymore myoepithelial cells. (**C**, **E**, **G**) – Expression of K-14, CK8&18 and SMA respectively in non-cultured human breast tissues. (**D**, **F**, **H**) – Expression of K-14, CK8&18 and SMA in breast explants cultured with 10 nM E_2 _for 21 days. (Scale bar, 100 μm).

Next we investigated how culturing conditions and the presence of different steroid hormones affect the structural components of the human mammary gland. For this purpose we chose markers for luminal and myoepithelial cells. Immunostaining for Cytokeratin 8&18 (CK8&18), Keratin-14 (K-14) and Smooth Muscle Actin (SMA) were used to investigate the effect of various hormones on the structure and differentiation of cultured human breast explants (Fig. 4A–H). CK 8&18 is a marker for luminal epithelial cells (LEC) and SMA for myoepithelial cells (ME). K-14 is a ME marker in normal tissue, whereas in transformed tissues it is regarded as a "proliferation" antigen (Fig. 4A,B).

Expression of CK8&18 and K-14 in explants were detected on days 0 and 21 of the experiment. Compared with non-cultured samples from the same patient, the immunostaining for CK8&18 was slightly declined after 21 days in the control, MPA and E_2_+MPA groups. A moderate intensity of CK8&18 immunostaining was observed in the E_2 _group (Fig. 4F). The immunostaining of Keratin-14 in E_2 _group after 21 days of culture showed that the epithelial cells positive for this marker were scattered throughout the acini (Fig. 4D) and in control group only ME were stained.

Immunostaining of SMA was found in the myoepithelium and stroma as well. The staining decreased markedly from day 0 to day 21 in the E_2 _group compared to control (Fig. 4H). In other treatment groups, the staining decreased slightly compared to control, but less than in E_2 _group.

### Assessment of Ki-67 and PCNA expression in cultured breast tissues

**Figure 5:**
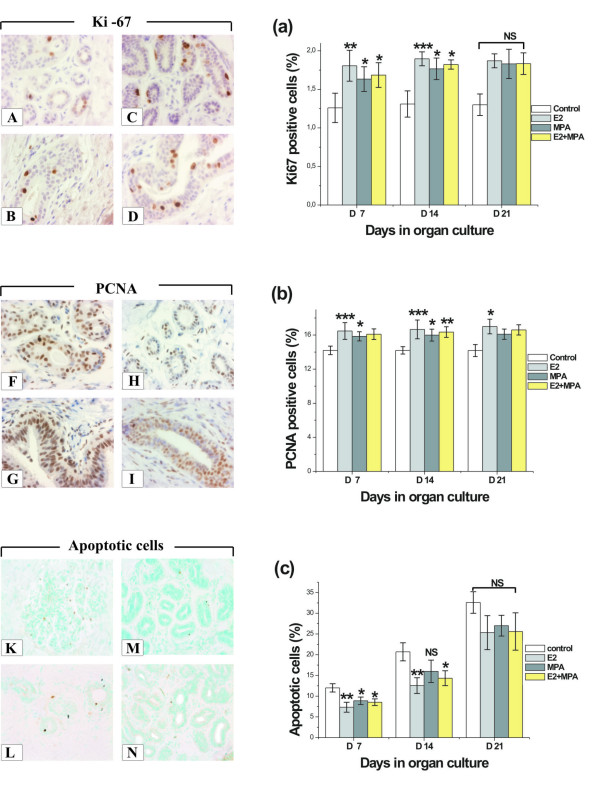
**Proliferation and apoptosis in human breast explants**. Proliferation in human breast explants (n = 9) at various time points and treatment groups was determined by Ki-67 and PCNA, and apoptosis by FragEL™. Comparison of expression of Ki-67 positive cells in explants cultured for 7 days (**A**-acini, **B**-duct) with explants cultured for 14 days (**C**-acini, **D**-duct) *in vitro *with E_2. _Note that cell proliferation was increased after 14 days *(p < 0.001)*. Examples of expression of PCNA in E_2 _treatment groups after 7 (**F**-acini, **G**-duct) and 14 days of culture (**H**-acini, **I**-duct). Note that intensity of PCNA staining diminished after 14 days when compared with staining intensity after 7 days of culture. Cell apoptosis in cultured explants after 21 days: (**K**)-control group; (**L**)-10 nM E_2 _group; (**M**)-100 nM MPA; (**N**)-10 nM E_2 _and 100 nM MPA group. The number of apoptotic cells decreased in all hormone treatment groups compared with control at 7, 14 and 21 days *in vitro*. Proliferation (*Ki67*, **a**, and *PCNA*, **b**) and apoptosis ratios (*FragEL*™, **c**). Proliferation and apoptosis indices were counted per 1000 epithelial cells at days 7, 14 and 21 of organ culture with different hormonal treatments. Columns represent the mean counts ± SD. Asterisks show the statistical significance of differences between control and hormonal treatment groups (*, *p *< 0.05}; **, *p *< 0.01}; ***, *p *< 0.001), *NS *not significant.

Ki-67 and PCNA were studied immunohistochemically to investigate the effects of different culture conditions on cell proliferation. Expression of both proliferation proteins was detected on days 7, 14 and 21 of culture. The percentage of epithelial cells detected with the anti-PCNA antibody was significantly greater than that detected with the Ki-67 antibody (Fig. 5A–I).

#### Immunostaining intensity

Immunostaining of Ki-67 was seen mainly in epithelial cells, but PCNA staining was detected in both epithelial and stromal cells. The staining intensities of PCNA and Ki-67 were estimated to be strong after 7 days of culture with E_2 _and moderate in the MPA and E_2_+MPA groups. In basal culture medium, intensity was estimated as moderate at 7 days and weak at day 21. In the E_2 _and E_2_+MPA groups from day 7 onwards, there were more PCNA and Ki67 positive cells in ductal epithelium compared to acinar epithelium. In control groups, TDLU and ducts stained similarly for Ki67 and PCNA at days 7, 14 and 21 days of organ cultures.

#### Proliferation indices

Proliferation indices (PI) were calculated on the basis of immunostaining of epithelial cells for Ki-67 and PCNA. The percentages of Ki-67 and PCNA positive cells per 1000 common epithelial cells in explants were expressed as PI for Ki-67 and PCNA. PI increased during organ culture in the presence of E_2_, MPA and E_2_+MPA.

PI for Ki-67 in the explants cultured in the presence of E_2 _was significantly greater when compared to control group at days 7 (*p < 0.01*) and 14 (*p < 0.001*) of the organ culture, but after 21 days of culture, the difference was not statistically significant. At 7 and 14 days other treatment groups as MPA and E_2_+MPA showed also significant differences (*p < 0.05) vs*. control group (Fig. 5a). Results with PCNA were comparable to the Ki-67 results, except that the difference between the E_2 _*vs*. control groups reached statistical significance (*p < 0.05) *after 21 days (Fig. 5b). Although the mean of PI values for the E_2 _group were highest after 7 and 14 days of culture, there were no statistically differences among the treatment groups E_2 _*vs*. MPA, E_2 _*vs*. E_2_+MPA or MPA *vs*. E_2_+MPA.

### Estimation of the rate of apoptosis

Apoptosis was observed mainly in epithelial cells. Steroid hormone addition to the culture medium affected apoptosis ratio *in vitro*. There were less apoptotic cells in all treatment groups (E_2_, MPA and E_2_+MPA) when compared to the control group (Fig. 5K–N).

The lowest apoptosis indices were found in the E_2 _treatment groups after 7 (*p < 0.01*), 14 (*p < 0.01*) and 21 (*NS*) days of culture compared to the control group. The difference between E_2_, MPA and E_2_+MPA *vs*. control groups did not any more reach a statistical significance after 21 days (Fig. 5c).

### Expression of ERα, ERβ and PR

**Figure 6:**
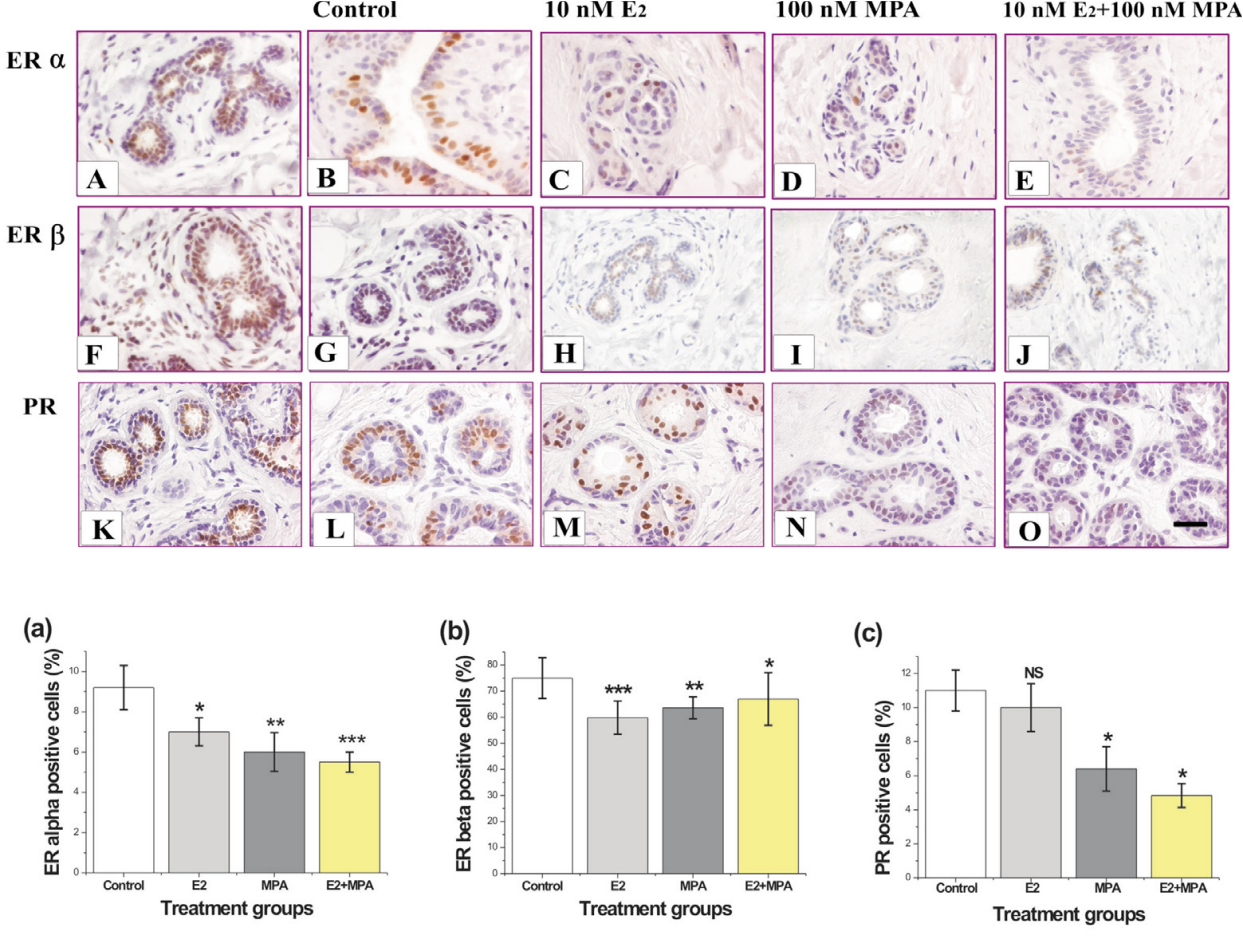
**Expression of steroid receptors in human breast explants**. Immunohistostainings of the steroid hormone receptors – ERα (**A-E**), ERβ (**F-J**) and PR **(K-O**). The effects of various hormonal treatments on the immunohistochemical expression of steroid hormone receptors – ERα (**a**), ERβ (**b**), PR (**c**). Columns represent the mean counts of positively stained cells ± SD. Asterisks indicate the statistical significance of differences between control and hormonal treatment groups (*, *p < 0.05*; **, *p < 0.01*; ***, *p < 0.001)*, *NS *not significant. (Scale bar, 100 μm).

Effects of different hormone treatments on expression of ERα, ERβ and PR were studied next. Non-cultured tissue samples were first immunostained for ERα, ERβ and PR. Only explants prepared from positive specimens were stained after culture period (Fig. 6A–O). Expression of ERα and ERβ was found in 67% and 100% of non-cultured samples, respectively. On average, ERα and ERβ labelling slightly decreased after 21 days of organ culture, compared to non-cultured samples or the control group at day 21 of culture. In general, all used hormonal treatments seemed to be associated with down-regulated expression of ERα (Fig. 6a) and ERβ (Fig. 6b). Expression of PR was found in 56% of the non-cultured samples. A strong positive immunostaining was observed for PR in explants cultured with 10 nM E_2 _substitution compared with MPA and E_2_+MPA groups. In the MPA and E_2_+MPA treatment groups, no PR or very faint staining for PR was detected (Fig. 6N,O).

## Discussion

Our results demonstrate that human mammary gland explants can be maintained viable in an organ culture system for 3 weeks without a major loss of tissue architecture. The results also show that addition of E_2_, MPA or combination of E_2 _and MPA to culture medium increased the rate of epithelial proliferation and decreased the apoptotic ratio. In addition, expression of estrogen and progesterone receptors was down regulated during these hormonal treatments *in vitro*.

Human breast tissue explants cultured by this method retained their morphological integrity. The presence of all tissue components in this model allows the investigation of the interactions between epithelium and stroma which have been demonstrated to be important for maintenance of the differentiated state of breast epithelium [22-25]. The morphology of explants cultured in basal culture medium without additions showed viable tissue with low acinar and ductal epithelium. Cultures in the presence of E_2 _and MPA or combination of both of them caused stimulatory effects on acinar and ductal epithelium. The number of epithelial cells increased, epithelium was layered and its height was elevated. Expression of differentiation markers was also affected by addition of various steroid hormones in the culture medium. The explants treated with E_2 _for 21 days showed decreased intensity staining for SMA, CK8&18 and K-14 positive epithelial cells scattered throughout the acini. The altered pattern of expression of differentiation markers along with the overall hyperplastic and slightly disorganised epithelial morphology suggests E_2 _decreased the level of differentiation of glandular epithelium *in vitro*.

Histomorphometry was performed on cultured explants to determine whether hormonal treatment affected epithelial parameters. We measured two morphometrical variables – acinar and ductal wall thicknesses. Histomorphometrical analysis revealed that the relative areas of glandular tissue components (acini and duct) increased among treated groups. Corresponding histomorphometrical analysis was done on human and macaques mammary gland treated *in vivo *with conjugated equine estrogen (CEE) with or without MPA [24,25]. These studies demonstrated that glandular tissue areas were greatest in CEE+MPA group. The results are contrary to ours, because we found that the highest epithelial thickness was in the explants treated with E_2 _only.

We also found that E_2_, MPA and their combination increased epithelial proliferation. In addition, Ki67 and PCNA proliferation indices for E_2 _group were constantly higher than for MPA or E_2_+MPA groups, although the differences between the treatment groups did not reach a statistical significance.

A wide spectrum of various methods has been used in recent years to identify proteins that are responsible for regulation of cell proliferation. Anti-PCNA and Ki-67 antibodies detect two different proteins present in cycling cells and have both been widely used to measure levels of proliferative activity in human tissues [34].

*In vivo *studies have reported conflicting results regarding proliferative activity in breast epithelial cells based on Ki67 and PCNA immunostainings. However, others and our study have demonstrated that E_2 _stimulated breast epithelial cell proliferation more pronounced than MPA or E_2_+MPA. *Foidart et al*. showed in a prospective study (where postmenopausal women received topical treatments with gels containing placebo, E_2_, progesterone (P) or E_2 _+ P applied directly to their breast for two weeks) that E_2_-alone increased the PI (PCNA) of breast epithelium 100-fold, P-alone 15-fold and E_2_+P combination gave an 13-fold increase [35]. Our *in vitro *results are in accordance with these observations. We observed proliferative response to hormonal treatment, especially with E_2_, similar as it was shown by *Anderson et al*. [36,37]. In their *in vivo *study pieces of human mammary gland were transplanted onto immune-compromised mice, which subsequently were treated with E_2 _and progesterone at the adequate doses of follicular or luteal phase of woman's menstrual cycle. They found that luteal phase concentration of E_2 _stimulate epithelial cell proliferation in normal human breast xenografts. Herewith, progesterone treatment alone had no effect compared to no treatment.

Other studies reported that oral E_2_-alone HT was associated with a smaller effect than E_2_+MPA on cell proliferation and epithelial density [20,31]. Some of the controversies between our results *in vitro *and *in vivo *findings can possibly be explained by differences in routes of administration and dosages of steroid hormone used. The route of administration influences the pharmacology of exogenous sex hormones. After oral administration, sex hormones are metabolised in liver and the intestinal tract to less active metabolites. When topically administered, peripheral aromatisation in fat and breast tissues may occur. Consequently, the concentrations in blood serum and target tissues may be different. When hormones containing gel was applied directly on breast skin, a high tissue concentration of E_2 _and the highest PI were obtained [35].

*In vitro *sex hormones are directly added to the culture medium and they affect the cells and tissues directly without any systemic effects. However, the effective concentrations may differ from physiological ones. In comparison with range of E_2 _in blood serum (around 2 × 10^-10 ^M) we have used a higher concentration, because the short-term presence of high concentrations and the long-term presence of low concentrations of estradiol cause a similar expression of estrogen-dependent products during a time interval of 12–48 h [38]. The clinically relevant blood concentration for MPA used for HT is 4 × 10^-9 ^M to 4 × 10^-8 ^M, but in a previous *in vitro *study lower concentrations did not produce any response [15,16].

It should be noticed that human breast is one of the few organs of the body that is not fully developed at birth. The full differentiation of mammary gland is reached only during pregnancy and lactation, by influence of specific hormones [39]. There are differences in histological structure of TDLU at various ages of woman's lifespan, which represent different stages of mammary gland development [39,40]. Four lobule types have been known so far. The lobule type 1 is the most undifferentiated structure, which is mainly found in postmenopausal breast. Lobule type 1 is associated with the highest level of cell proliferation, decreasing progressively in lobule type 2, 3 and 4 [39,41]. We cultured postmenopausal women's breast tissues containing mostly undifferentiated epithelial cells, which have a high capacity to proliferate by influence of various endogenous and exogenous stimuli such as steroid hormones. We found an intensive proliferative response and, consequently, an increased epithelial density in hormonal treatment groups.

It is known that hormone-induced cellular proliferation can alter susceptibility to carcinogens or mutogens [42]. Uncontrolled cell proliferation is considered a hallmark of tumorigenesis. Our results showed that besides proliferation also apoptosis ratios were dependent on hormonal treatments. Especially treatment with E_2 _decreased apoptosis ratio in the epithelial cells as has been shown in breast cancer cells [43].

The effects of E_2 _and P on the mammary gland are mediated by their nuclear receptors – ER and PR [44]. Two forms of ER, namely ERα and ERβ, encoded by different genes, are known [45]. Progesterone receptor also has two isoforms – PRA and PRB, which are coded for a single gene. ER and the two isoforms of PR have different distribution in human breast [46]. ERα is expressed at a low level only in luminal epithelial cells of resting mammary gland (~ 10–20%). During lactation 80% of the luminal cells express ERα [46]. ERβ is expressed not only in luminal epithelial, but also in myoepithelial and stromal cells. ERβ is dominant in the resting mammary gland and its levels are high in normal breast tissue (~ 70%) [47]. PR has been shown to be present, like ERα, only in luminal epithelial cells (15–30%) [48]. Using the dual-label immunofluorescent techniques, an almost complete co-expression between ERα and PR (96% of the cells expressing PR also contain ERα) has been reported [48].

In normal breast tissue only differentiated cells are considered to express ERα or PR and they do not proliferate [44]. Undifferentiated cells (the stem cell/early transit cell populations) are capable to proliferate [49]. Almost every proliferating cell is adjacent to one expressing ERα and PR [49]. Therefore it is possible that ERα and PR-positive cells control the proliferation activity of undifferentiated cells *via *juxtacrine and/or paracrine signals depending on the prevailing estrogenic environment [50].

We found that the relative numbers of ERα, ERβ and PR positive cells in the presence of steroid hormones were decreased after 21 days of culture, especially ratios of PR positive cells in MPA and E_2_+MPA groups. Generally, steroid hormones regulate the expression of their own receptors. During the menstrual cycle, as E_2 _levels increased, ERα is down-regulated, whearas ERβ is up-regulated [51]. These results are consistent with our finding that in E_2 _treated explants, ERα expression in epithelial cells is decreased. To the contrary previous study, we found that expression of ERβ decreased in the presence of steroid hormones. Both *in vitro *and *in vivo *experiments have indicated that ER beta exerts an antiproliferative effect on human breast cells [52]. ERβ expression is relatively high in the normal breast, which is inversely correlated with cellular proliferation. There are some data showing that ERβ expression is downregulated in lesions such as atypical ductal hyperplasia and DCIS when compared with that in normal epithelium. Although reduced, invasive breast carcinomas show high levels of ERα and ERβ with approximately two thirds of the tumors staining positive by immunohistochemistry [53]. These elevated expressions of ERs are associated with dysregulation of proliferation (increased numbers of proliferating cells) [6].

Our results showed a proliferative response to exogenous E_2 _and progestin treatment, which was accompanied by a decrease in the number of ERβ cells. Steroid hormones suppressed the ratio of ER and PR positive cells, which are though to be the most differentiated ones that control the proliferative activity of less undifferentiated epithelial cells in breast tissue.

Many studies have shown that progesterone down-regulates its own receptor and antagonizes the estrogen action by inhibiting the synthesis of ERs [54,55]. Our results show an unexpected E_2 _reduction of PR in epithelial cells of breast explants. This result contradicts the original report made by *Anderson et al*. that E_2 _upregulated PR expression in human mammary gland xenografted into athymic nude mice [36,37].

There are also previous reports on a complete dissociation between the level of steroid receptor (ERα and PR) expression and proliferation rate in normal human mammary epithelium [6]. Our results showed that postmenopausal breast tissue *in vitro *responded to exogenous stimuli (hormonal treatment) with intensive proliferative response associated with a decreased relative number of ERα, ERβ, PR positive cells. We observed the inverse relationship between steroid receptor expression and proliferation in our *in vitro *model (proliferation ratios increased and simultaneously the number of ER, PR positive cells decreased). On the other hand, a loss of inverse relationship between steroid receptors and proliferation rate was reported to occur at the earliest stages of breast tumorigenesis (increasing the number of proliferating cells with increase of expression of ER, PR) [50]. Dysregulation of the relationship between steroid receptor expression and proliferative activity in our cultures may also be associated with the fact that the tissue samples were obtained from breast tumour patients even if the histology of explants was normal. Even so they provide a valuable model for studies on the effects of E_2 _and progestin treatment on epithelium of postmenopausal mammary gland and the mechanisms of dissociation of hormone regulation of receptor levels and proliferation.

Taken together, it seems that *in vitro *the postmenopausal breast tissues possess mostly undifferentiated epithelial cells [39], which respond to exogenous stimulus produced E_2 _but also progestin and their combination treatment with a proliferative response and decreased number of ER and PR positive cells.

## Conclusion

The present study indicates that organ culture provides a vitro model in which architecture of human mammary gland can be maintained. The results obtained by using organ cultures demonstrated direct effects of E_2 _and MPA on the proliferation and differentiation on human breast tissue without the interference with the systemic effects found *in vivo*.

It is well-known that estrogens and/or progestogens can influence proliferation either directly by regulating the cell cycle [56] or indirectly by regulating production of stimulating or inhibiting stromal or epithelial growth factors [15,57]. Thus, organ cultures in many aspects provide a more appropriate model for estimation of the proliferative effects of various factors and compounds than cell cultures. The advantage of this method is that the proper connection between stromal and epithelial cells is maintained.

## Abbrevations

ET = Estrogen replacement therapy;

HT = Hormone replacement therapy;

E_2 _= Estradiol;

MPA = Medroxyprogesterone acetate;

H&E = Hematoxylin/eosin;

ER = Estrogen receptor;

PR = Progesterone receptor;

P = Progesterone;

CK = Cytokeratin;

K = Keratin;

SMA = Smooth muscle actin;

LEC = Luminal epithelial cells;

ME= Myoepithelial cells;

PI = Proliferation index;

## Competing interests

The author(s) declare that they have no competing interests.

## Authors' contributions

RE, the principal investigator, conceived the study and supervised the project. PH identified the proper approaches to evaluate influence of steroid hormones on human breast *in vitro*. NE performed organ cultures, immunohistochemistry and drafted the manuscript. RE, PH and NE interpreted the data. RE and PH revised the manuscript. All authors read and approved the final version of the manuscript.

## Pre-publication history

The pre-publication history for this paper can be accessed here:



## Figures and Tables

**Table 1:** The monoclonal primary mouse antibodies used in this study.

**Primary antibody**	**Company**	**Antibody dilution**	**Staining pattern**
Actin, Smooth Muscle Ab-1	NeoMarkers, Fremont, USA	1:400	Cytoplasmic
anti-Cytokeratin 8&18	Zymed® Laboratories Inc, South San Francisco, CA	1:250	Cytoplasmic
anti-Human Estrogen Receptor – alpha	DakoCytomation, Glostrup, Denmark	1:2000	Nuclear
Anti-PCNA	Zymed® Laboratories Inc, South San Francisco, CA	1:200	Nuclear
Estrogen Receptor – beta	Novocastra, Newcastle Upon Tyne, UK	1:800	Nuclear
Keratin 14 Ab-1	NeoMarkers, Fremont, USA	1:400	Cytoplasmic
Ki67 Antigen	Novocastra, Newcastle Upon Tyne, UK	1:100	Nuclear
Progesterone Receptor (A/B Forms)	Novocastra, Newcastle Upon Tyne, UK	1:100	Nuclear
